# Implementing change in primary care practice: lessons from a mixed-methods evaluation of a frailty initiative

**DOI:** 10.3399/bjgpopen18X101421

**Published:** 2018-04-07

**Authors:** Carol Bryce, Joanna Fleming, Joanne Reeve

**Affiliations:** 1 Research Fellow, Warwick Primary Care, Warwick Medical School, Coventry, UK; 2 Research Fellow, Warwick Primary Care, Warwick Medical School, Coventry, UK; 3 Professor of Primary Care Research, Academy of Primary Care, Hull York Medical School, Hull, UK

**Keywords:** frailty, primary care, implementation, Normalisation Process Theory, NoMAD

## Abstract

**Background:**

The NHS is facing increasing needs from an aging population, which is acutely visible in the emerging problem of frailty. There is growing evidence describing new models of care for people living with frailty, but a lack of evidence on successful implementation of these complex interventions at the practice level.

**Aim:**

This study aimed to determine what factors enable or prevent implementation of a whole-system, complex intervention for managing frailty (the PACT initiative) in the UK primary care setting.

**Design & setting:**

A mixed-methods evaluation study undertaken within a large clinical commissioning group (CCG). Design and analysis was informed by normalisation process theory (NPT).

**Method:**

Data collection from six sites included: observation of delivery, interviews with staff, and an online survey. NPT-informed analysis sought to identify enablers and barriers to implementation of change.

**Results:**

Seven themes were identified. PACT was valued by professionals and patients but a lack of clarity on its aims was identified as a barrier to implementation. Successful implementation relied on champions pushing the work forward, and dealing with unanticipated resistance. Contracts focused on delivery of service outcomes, but these were sometimes at odds with professional priorities. Implementation followed evidence-informed rather than evidence-based practice, requiring redesign of the intervention and potentially created a new body of knowledge on managing frailty.

**Conclusion:**

Successful implementation of complex interventions in primary care need inbuilt capacity for flexibility and adaptability, requiring expertise as well as evidence. Professionals need to be supported to translate innovative practice into practice-based evidence.

## How this fits in

Transformation of primary care services is needed to respond to the changing needs of an aging population living with complex problems. Complex problems need complex interventions, supporting practice-level change in service design and delivery. As yet, evidence on and understanding of successful implementation of these interventions at the organisational level is lacking. This study describes the critical evaluation of a new complex intervention to address frailty within a UK primary care population. These findings reveal evidence of three new factors needed to support transformation in this locality. The next step is to examine whether these apply across the wider primary care context.

## Introduction

The challenge facing the NHS is to deal with the mismatch between the needs of an aging population living longer with chronic, complex illness,^1^ and a health service not configured to meet the growing and changing demands.^2^ It is recognised that doing more of the same will not solve the problems.^3^ There are national and international calls for health service redesign, away from the management of disease to supporting 'whole person' and person-centred care.^4–6^

These challenges are acutely visible in work to address the emerging problem of frailty.^7^ Frailty describes a diminishing capacity to recover from ill health.^7^ People living with frailty are vulnerable to problems of both overtreatment (by virtue of reduced capacity to manage the work of medical care and treatment) and of underdiagnosis or undertreatment (by virtue of healthcare prioritisation focusing on different priority areas to patients' needs). Aging is a risk factor for frailty. Tackling frailty is a priority area for health services nationally and locally.

A growing body of evidence describes best practice in the management of frailty. Guidance offers tools to help identify people at risk, to assess health needs, and to manage care.^8^ The challenge for local health teams is now to translate guidance into services on the ground. A recent report from the Royal College of General Practitioners (RCGP) describes a number of case studies of successfully established service development projects.^9^ The authors identify some common themes that seemed to contribute to success, including the need for professional development, collaborative multidisciplinary working, the importance of patient engagement, and the need for investment and resources.

The innovations described within these frailty case studies are examples of complex interventions; that is, interventions with multiple interacting components. Frailty is a ‘whole person’ concept of illness and health need. As such, it requires care approaches that include many elements, flexibly applied to meet the varied and varying needs of individuals. Design, implementation, and evaluation of complex interventions all bring their own challenges, but each also offer opportunities to critically understand and influence service development and improvement.

A growing body of research examines implementation of complex interventions. In a recent 'systematic review of reviews' of this work, Lau and colleagues noted a 'substantial literature' looking at interventions aimed at changing specific professional behaviour or practice.^10^ From this, they identified a number of features potentially associated with successful implementation, themes which resonate with the RCGP report findings (for example having clear goals, involvement of stakeholders, and appropriate resources); but they also noted a lack of evidence describing what organisational-level structures and processes are needed to implement new ways of working at a practice level.

This article describes an evaluation of the implementation of a frailty initiative in a UK primary care locality. The locally-named PACT initiative was a pragmatic response of a local health system to meet the needs of its population. Local commissioners issued guidance describing evidence-based tools to support delivery of care to people living with frailty, and commissioned local GP practices to implement the new service. The present authors undertook a critical evaluation of the implementation of this initiative, aiming to determine what factors enable or prevent implementation of a whole-system, complex intervention for managing frailty in the UK primary care setting.

## Method

The study was a mixed-methods implementation evaluation study, informed by NPT^11^ (Box 1). The study site was a large CCG in the Midlands, UK. The location has a population that is slightly more rural, has fewer ethnic minorities and unemployed individuals, and a larger older population than the rest of the Midlands or UK.^12^ The site was divided into six localities each responsible for implementing the PACT initiative in their own area. Each locality consisted of one PACT team (with variable professional membership), working with a range of 4–11 GP practices. All localities were invited to take part in the evaluation; five accepted. At each locality, staff were recruited from the PACT teams and the local practices to take part in the evaluation.

**Box 1.** The four domains of work in normalisation process theory

Normalisation process theory predicts that for any new way of working to become fully embedded into everyday practice needs continuous work in four areas (domains) of activity:^11^	
**Domain**	**Description of work involved**
**Sense making**	The new intervention must make sense to the people responsible for implementing it, including that they recognise it as a distinct and different way of working.
**Engagement**	People must choose to engage with the new way of working, including those who lead or pioneer the introduction, along with the majority choosing to join.
**Action**	People need the resources to implement the new way of working, whether that be skills, time, or other.
**Monitoring**	People must get feedback on the new approach; feedback which encourages them to keep going.

Data collection explored enablers and barriers to implementation of PACT. Three approaches (Box 2) were used, including researcher observation of PACT delivery; interviews with a purposive sample of key staff involved in implementation and delivery; and an online survey of all staff from across the five localities using the NoMAD tool.^11^ NoMAD is an NPT-informed, 23-item instrument for measuring implementation processes from the perspective of professionals directly involved in the work of implementing complex interventions. Observations and interviews were with staff directly involved in delivering the PACT service. NoMAD extended data collection to include staff from across the primary care setting, including clinical, management and commissioning staff.

**Box 2.** Detailing data collected

	﻿
**Observation of PACT delivery**	Shadowing members of the PACT team at each location for half a day, including mini interviews with staff and patients.
**Interview data**	6 individual GP interviews, and a focus group with 6 members of a local PACT team.
**Online survey using a study modified version of the NoMAD tool**	The qualitative data were used to support the development of a bespoke version of the NOMAD evaluation tool (a survey tool developed from normalisation process theory to assess the implementation of new initiatives in practice). The survey was sent to all staff in the five sites, with 90 responding (45% response rate) including 39 GPs, 11 PACT nurses, 12 other primary care nurses (including district and practice nurses), 2 care home practitioners, and 22 practice managers, team managers, or administrators.

The study team opted not to collect data from patients at this stage. This was partly for pragmatic reasons connected to resources available, but also reflected a primary focus on understanding the experiences of staff in delivering evidence-based innovation on the ground.

Analysis followed a modified framework approach developed in the authors' previous NPT-informed implementation studies.^13^ The observational and interview datasets were initially examined to identify examples of enablers and barriers to implementation of the new frailty initiative, using the four areas of work described by NPT (Box 1) as a sensitising tool, but still allowing for other themes to emerge. Data were coded by one researcher, with a proportion of the data also coded by a second researcher. Coding was compared and any disagreements were discussed before further analyses took place. The emerging framework was used to analyse the full dataset including the NoMAD survey data. Constant comparison was used to synthesise the emerging descriptive codes into seven explanatory themes.

## Results

A full descriptive analysis of the individual datasets is included in the final study report, which is available from the authors on request. The analysis presented here focuses on the emerging themes from across the dataset that describe and explain identified barriers and enablers to innovation and implementation. It should be noted that selected quotes used within this report are representative of the wider dataset.

Seven themes were identified from the dataset summarised in Box 3, and these are discussed below.

**Box 3.** Summarising key themes emerging

NPT themes	Themes emerging from the data	Why this matters	Examples from dataset
**Sense making**	Valued and of value	Core to professional values and patient values (driver that keeps things going in face of adversity).	52% see potential value of PACT to their professional role (NoMAD) *‘I think you know, we've come as a practice … to recognise the value I think, let's put it that way.’* (GP interview)
	Lacks clarity	Lack of clarity in aim and purpose led to differing implementation across the location.	21% do not believe staff in their organisation share understanding of the purpose of PACT (NoMAD) *‘The problem is, because they’re working across different practices, everybody had different ideas of what they wanted them to do.’* (GP interview)
**Engagement**	Need champions	To drive the initiative forward and to adapt working practices.	51% agree there are key people driving PACT (NoMAD). 88% agree that they are open to working with colleagues in a new way to make PACT work (NoMAD). *'I am desperate to try and improve the* [usual] *service … I cannot agree with the service continuing in its current form. I feel I am serving a bureaucratic service which only benefits some of the patients some of the time.'* (PACT nurse) *‘I’m a big believer that it depends on the person doing the role from the PACT team, and what they are doing.’* (GP interview)
**Action**	Redesign required, not plug-in of evidence-based frailty tools.	Necessary because of variability in need; variation in understanding; to overcome disruption and fragmentation; and to establish new, or add missing, infrastructure.	*‘ ... people will acknowledge that the tools available haven't been that good.’* (PACT GP)
	Unanticipated resistance	Recognising additional, unanticipated patient resistance that required work. Families also have to be included in that work.	33% of staff had confidence in patients’ ability to use PACT (NoMAD). *‘We’ve had the odd patient who doesn’t want it, who is a bit like, they don’t want it … "Oh no, I’ve always had the doctor, I want the doctor to come and do that." '* (PACT GP)
**Monitoring**	Service-focused outcomes	Monitored outcomes differed from the reported motivators and drivers for doing the work (professional and patient-centred values).	33% agree that they have received feedback about PACT (NoMAD). *‘What we should be doing is saying, "Well we're just going to put all the money into this, get a good service up and running because we know it's the right thing to do"* [but]* … the outcome measure really is a reduction of inappropriate admissions, but you will never be able to measure that because of the context of A&E demands and all the rest of it.’ (*PACT GP)
	Generating new knowledge	Vital to making the implementation work. Mechanism to generate practice-based evidence. Help healthcare professionals recognise and trust quality that is beyond guideline.	90% agree that feedback about PACT could be used to make future improvements (NoMAD). *‘… it was all a bit on the back of an envelope to be honest. But essentially … we all had a list of maybe five or six people we knew who were frail. We combined that with the top 2% and then the people who were going into hospital frequently and we've got a list, essentially.’* (PACT GP)

### Valued and of value

It was evident that most staff valued the frailty work, which resonated with core professional values of offering patient-centred care. Members of PACT teams also felt the service was of value based on the feedback from their individual patients, most of whom welcomed the new service. This support was important to enable the leaders of the new service to mobilise and engage others in participating in the implementation of the frailty initiative.

However, there was also some disconnect between the views of staff delivering PACT, and those working in the localities but not directly involved. These staff welcomed the additional resources PACT offered to expand service capacity for a ‘needy’ group. However, they didn’t recognise innovation within PACT or see it as offering anything new or different than standard care. They were more questioning of the value and purpose of the service as an ‘innovation’.

### Champions driving the work

All responders recognised the daily work of PACT as complex, creating a busy and demanding job for those involved in delivering the service. Many people were contributing to the work, under the direction of key leaders at each site. Each locality revealed needing the presence of a champion or champions to drive the service development and implementation. Champions differed across sites in terms of professional role. In some places, GPs were the driving force; in others, PACT nurses. Champions described needing to develop and use skills in adapting and developing the service, rather than specific clinical skills. The importance of skilled local champions was highlighted by localities where this was missing (for example, due to staff sickness). These sites struggled to adapt and evolve in response to changing demands on the service.

### Unanticipated resistance

In some localities, staff described examples of suspicion and resistance to change from patients and, to a lesser extent, their families. For example, people wanted to continue to see 'their GP' rather than a new team. Staff described having to spend time explaining to some patients what the new service was for and why it had been introduced. They reflected that the service had not been developed with, or advertised to, local residents prior to its introduction.

### Lack of clarity of purpose

While staff from across the dataset consistently welcomed additional resource for, and a shift to, person-centred care for a vulnerable community, they also highlighted uncertainty about the specific remit of the new service. This lack of clarity in defining the boundaries of the new initiative left staff feeling unclear about where responsibility ended, both in terms of accountability for clinical decisions, and in defining the limits of care (for example, in addressing social needs such as loneliness). Ongoing uncertainties limited further implementation; for example, with staff time being taken up chasing ‘bottomless’ details for patients already in the service, and so being unable to accommodate new patient needs.

### Service-focused outcomes

The national guidance used to support implementation focuses on an outcome of reducing unplanned admissions. This translated into contractual targets for local services. Yet this study's data highlighted that these contractual priorities differed from factors that motivated staff to develop and deliver a complex new way of working, including professional values (supporting person-centred care) and patient-focused outcomes (for example, improvements in daily living). Staff consistently described the heavy workload on PACT teams, with motivated staff needing to go 'above and beyond' to deliver care. The impact, if any, of this mismatch in priorities between contract and staff motivation on service sustainability had yet to be revealed at the time of writing. However, it was apparent that uncertainty over boundaries of care (how far to go in identifying and seeking to address personal needs) was creating tension and burden for staff.

### Redesign, not a bolt-on

Data highlighted that the PACT innovation was not a ‘bolt-on’ service change, a ready-made package that could be instantly delivered by a suitably staffed team. Rather, implementation involved adaptation and amendment on the ground to develop a service model to suit local needs.

This analysis revealed that the evidence-based tools described in local (PACT) and national guidance needed reshaping to meet local (perceptions of) needs. For example, staff reported that frailty identification tools both over- and under-diagnosed need, with staff commonly returning to the use of clinical judgement to create lists of frail vulnerable patients who should be offered the new service. The geriatric comprehensive assessment tool was also described as needing to be adapted to suit local preferences and experiences of use. Some used shortened forms of the tool; others abandoned it and reverted to clinical acumen. Much of the data described the work to adapt ‘evidence’ to ‘clinical need’. Many PACT staff expressed frustration at being 'left to get on with it' without support, describing the initial support and enthusiasm from commissioners as short-lived, leaving them to struggle on. They also described that the developmental aspect of their role, needed to make PACT work, was above and beyond the contracted service. Significant levels of sickness absence in some teams were noted, although with insufficient data to describe a causal link.

### Generating new knowledge

The observation and interviews revealed a rich seam of developing professional expertise derived from critical reflection on implementation of evidence-informed practice. The evaluation revealed the generation of new, practice-based knowledge, but also that staff lacked the training and/or confidence in critically assessing the value of this knowledge, and so potentially undervalued their individual and collective learning from implementation.

## Discussion

### Summary

This article describes an in-depth examination of the implementation of a new primary care complex intervention at an organisation-wide level. From these findings, three key factors potentially necessary for implementation were identified: the capacity for flexibility and adaptability; the need for expertise, not just evidence; and the potential for redefining professional roles.

Flexibility and adaptability were necessary to translate evidence-based guidance into evidence-informed practice on the ground. Clinical pragmatism lay at the heart of much of the adaptation, driven by a collective sense within PACT teams of needing to ‘make it work’, to deliver on contractual targets, but also to recognise professional priorities and values (especially related to person-centred care) that mattered to the staff involved.

Flexibility brought additional unanticipated challenges in terms of defining the boundaries of the new service, with several staff commenting on the constant expansion of identified patient need threatening to overwhelm service capacity. The findings highlight the importance of establishing a shared statement of purpose, a framework that supports professionals to adapt implementation where appropriate, but also gives them permission to ‘say no’.

The need for expertise, not just evidence, was the second key finding. Evidence-based tools were insufficient to deliver individual care, or to implement the new service. The authors witnessed examples of new expertise developing within PACT teams, both in the critical development of clinical practice (through critical assessment and modification of models of care), and also in strategic expertise (evaluating the wider aim, purpose, and direction of travel of the new service). But staff also widely reported a mismatch between capacity and need in this respect. Service contracts — and so outcomes for which practices were paid — focused on delivering, not developing, models of care; but commissioners of care were not perceived to be providing this necessary leadership. There was evidence of staff seeking to fill the gap, but also revealing uncertainties about whether they had the capacity, skills, and oversight to deliver.

Implementation of this complex intervention required and revealed extended professional roles. Staff from multiple disciplinary backgrounds were actively engaged in service development work, applying, and actively developing, extended experience in critiquing and amending models of care. However, these extended roles were largely under-recognised and rewarded by contractual mechanisms. This gap between actions done and actions rewarded contributed to the described uncertainty about roles and boundaries, which undermined staff confidence in developing and continuing these roles. Failure to adequately reward staff for work done impacts on motivation and therefore the sustainability of the service.

### Strengths and limitations 

This was a theory-based, in-depth analysis of change on the ground. Triangulation across multiple datasets was used to add depth and trustworthiness to the analysis. These findings resonate with and extend previous research, particularly in focusing on the whole-system implementation of a new initiative. The present authors raise new hypotheses and suggestions for supporting sustained scholarly redesign of primary care. However, the study was limited to one location in the UK. The next step will be to repeat this work in other settings, to explore for consistency and variation across UK settings. Given the constraints of time and funding, an active decision was made with commissioners not to include patients in this initial evaluation, which focuses on frontline professional perceptions of service development. Future work should triangulate findings with the public and patients, as well as policy-maker perspectives.

Particularly during the observational phase of this work, the intention was to use NPT as a sensitising framework to help alert the authors to potential enablers and barriers, while remaining open to other areas also emerging. All of the data identified mapped to the NPT domains. It may be that other theoretical frameworks could flag additional themes. The present authors propose that future work should also consider including, for example, the theoretical domains framework for behavioural change. The latter focuses on understanding change at the individual behaviour level, rather than organisational levels. This may add valuable detail to some of the themes described by this dataset and analysis.

### Comparison with existing literature

These findings contribute to addressing the gap in the literature identified by Lau *et al* ^10^ in developing an understanding of complex intervention implementation at an organisational level.

Lau *et al* previously described the need to 'tailor' complex interventions to support successful implementation.^10^ In the studies cited in their review, tailoring involved the use of facilitators to ‘tweak’ an intervention in order to optimise local adherence. This is in contrast to the present study's findings, which recognise tailoring as a process of local staff working to critically evaluate the use and utility of components of the intervention (the PACT guidance). This results, in some cases, in major changes to, or even rejection of, elements of the intervention provided. There are inevitable challenges for evaluating ‘fidelity’ of implementation, including whether successful implementation is recognised in terms of adherence to component parts, or overall achievement of the core component of the complex intervention (in this case, addressing frailty). The need for clear, shared strategic goals for care is once again underlined.

The observation of PACT staff working to adapt evidence-based guidelines into evidence-informed practice resonates with Gabbay and le May’s previous description of the translation of guidelines into mindlines.^14 ^They described the process by which professionals work together to critically review, refine, and develop ‘evidence’ to meet local needs and perspectives, generating 'knowledge-in-practice-in-context'.^14^ Gabbay’s work focused specifically on the modification of clinical knowledge. This study extends these observations into the wider arena of professional practice, namely the development and delivery of care at the practice and organisational level.

Developing capacity for, and confidence in, extended professional roles is a key aim for a joint initiative between the Society for Academic Primary Care and the RCGP in their 'GP Scholarship' work.^15^ Aiming to champion and cultivate the intellectual expertise of professional practice, this programme of work seeks to support development of skills and opportunities for extended professional roles within general practice and the wider primary care clinical community.

### Implications for practice and research

These findings suggest a number of factors need to be considered when commissioning a new primary care innovation service. These include: the need for a sustained strategic lead responsible for clearly defining strategic aims, with an ongoing role providing oversight that supports confident adaptability; secondly, the need for dedicated expertise in the critical evaluation and development of practice-based evidence, supporting quality generation and confidence in application; and thirdly, the need for revised contracting mechanisms, which build in resource and monitoring that recognises, supports, and feeds back on the development as well as delivery of services.

These findings are based on an in-depth study of a frailty-based service development initiative in one location. Future research should examine how these findings apply to frailty initiatives in other settings, and to service development that doesn’t involve frailty, in order to develop guidance for commissioners on service innovation. It would be important to then evaluate the impact of introducing such guidance both on staff morale, as well as on service and patient outcomes. In this way, primary care redesign can be driven which is underpinned by the generation of practice-based evidence, thus supporting sustained and scalable change. 
